# Living at the Interface: Behavioral, Evolutionary and Ecological Insights of Spring Use by Highly Mobile Stygobiont Crustaceans, *Troglocaris planinensis* (Decapoda: Atyidae)

**DOI:** 10.1002/ece3.73338

**Published:** 2026-03-27

**Authors:** Raoul Manenti, Veronica Zampieri, Filippomaria Cassarino, Damiano Brognoli, Edgardo Mauri, Giorgia Terraneo, Elia Lo Parrino, Raffaele Bruschi, Valentina Balestra, Stefano Lapadula, Matteo Galbiati, Valeria Messina, Mattia Falaschi, Benedetta Barzaghi, Gentile Francesco Ficetola, Andrea Melotto

**Affiliations:** ^1^ Department of Environmental Science and Policy Università degli Studi di Milano Milano Italy; ^2^ Laboratory of Subterranean Biology “Enrico Pezzoli”, Parco Regionale del Monte Barro, Località Eremo, 1 Galbiate Italy; ^3^ Società Adriatica di Speleologia Trieste Italy; ^4^ Department of Chemical and Pharmaceutical Sciences University of Trieste Trieste Italy; ^5^ Department of Environment, Land and Infrastructure Engineering Politecnico di Torino Torino Italy; ^6^ Biologia Sotterranea Piemonte – Gruppo di Ricerca Frabosa Soprana (CN) Italy

**Keywords:** Atyid, cave, decapod, ecotone, groundwater, light, predator, shrimp, *Troglocaris*

## Abstract

Springs represent ecotones between groundwater and surface freshwater habitats. Recent research suggested that springs can be more important than expected for stygobiont (i.e., adapted to live in groundwater) species, still information on habitat exploitation and activity of stygobionts in springs is far from complete. The aims of this study are: (i) to identify environmental factors promoting the exploitation of ecotone habitats by the stygobiont shrimp *Troglocaris planinensis*, commonly found in spring environments in northeastern Italy; and (ii) to experimentally evaluate whether this species exhibits differential behavioral responses to light and to subterranean and surface predator cues based on its habitat of origin (spring versus cave). From June 2020 to January 2025, we started multiple day and night surveys of *T. planinensis* in 64 springs of the Classic Karst (NE‐Italy). Each site has been characterized with respect to abiotic and biotic features. In the laboratory, shrimps from both cave and spring populations were tested to assess behavioral differences in response to light stimuli and predatory cues, as potential adaptations to the contrasting conditions of their respective habitats. In springs, *T. planinensis* density reached up to 116 shrimps/m^2^, with significantly higher counts at night and lower densities at sites with greater fish predator abundance. Laboratory tests showed that predator cues, but not light exposure, influenced shrimp behavior regardless of their cave or spring origin. This study suggests that stygobiont crustaceans can represent a significant portion of biomass in surface waters and exploit these environments in response to changes in abiotic and biotic conditions and stimuli. However, further research is necessary to determine how stygobionts perceive surface conditions and how ecotonal pressures may drive adaptive shifts in typically groundwater‐dwelling animals.

## Graphical abstract

The study provides evidence that animals considered as strictly linked to subterranean habitats can exhibit behavioral responses to constraints occurring in surface environments. We studied the blind and depigmented shrimp *Troglocaris planinensis* in the Classical Karst using field and experimental approaches. In springs, stygobiont shrimps are often numerous, especially during night and without the pike, which is a top‐predator fish. In laboratory, shrimps respond to cues of surface (pike) and subterranean (olm, cave tetra) predators.

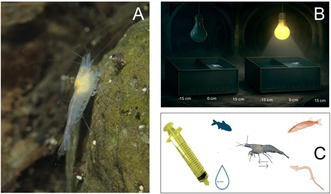

## 1 Introduction

The study of ecotones can be particularly intriguing for ecological research, as ecotones are habitats of transition between adjacent ecosystems for biological communities. Furthermore, ecotones can occur at different spatial scales and can range from transitions between natural ecosystems to human‐generated interfaces across strongly modified habitats (Kark 2013). Although boundaries are often assumed to represent clear‐cut divides between regions, habitats and communities, ecotones can allow the gradual transition between different environmental conditions and assemblages, generating a complex and variable mosaic of ecological pressures (Bowersox and Brown 2001; Gibert et al. 1997; Kark 2013; Luštrik et al. 2011; Prous et al. 2004). Various studies have shown that biodiversity and species abundances can show striking variation across ecotones (Ward et al. 1998; Williams 2003). Moreover, evidence suggests that ecotones can promote evolutionary processes, because adaptive shifts in response to the different constraints encountered in transitioning zones may be positively selected in populations exploiting the boundaries of their distribution (Schilthuizen 2000). As such, the study of ecotones can provide intriguing insights into the behavioral and ecological mechanisms driving adaptation of organisms facing novel selective pressures.

Among freshwater habitats, springs represent one of the most interesting ecotone. Springs essentially are the transition between groundwater and surface freshwater habitats (Alfaro and Wallace 1994). Environmental conditions and organisms of both underground and surface habitats interplay in characterizing spring features. While the contribution of surface species on spring communities is relatively studied (Falasco et al. 2012; Ilmonen et al. 2009), the role of groundwater organisms and the environmental characteristics of these habitats have received less attention (Di Lorenzo et al. 2018; Manenti, Forlani, et al. 2023). Along the groundwater‐surface transition, multiple biotic and abiotic factors co‐vary shaping the environmental features available to organisms (Von Fumetti and Nagel 2011). For instance, springs have a similar temperature and water chemistry to cave streams, but are subject to the daily variation of sunlight exposure, and receive resources from vegetation that are absent from the groundwater system. At the same time, while being more stable and less risky than surface streams, springs are exposed to wider microclimatic oscillations and to broader range of surface predators that venture in them, compared to groundwaters (Cantonati et al. 2021; Manenti et al. 2013). This gradient is also affected by diel variation, as during the day UV radiation represents a significant barrier which limits stygobionts (i.e., animals specialized to live in groundwater) from wandering toward epigean environments (Manenti et al. 2018; Manenti, Forlani, et al. 2023). These complex pressures may determine adaptations in subterranean organisms. For instance, depigmented and eyeless stygobionts of the genus *Niphargus* (Crustacea: Amphipoda) and stygobiont planarians of the species *Dendrocoelum italicum* are still able to respond to light stimuli (Barzaghi et al. 2025; Borowsky 2011; Fišer et al. 2016). This feature has been associated with the necessity to recognize and avoid surface habitats, but might also be viewed as a trait that enables them to take advantage of surface habitats at night, when UV radiation is absent and conditions are more favorable (Manenti and Barzaghi 2021). The abundance of predators is another important distinction between surface freshwater and groundwater. While surface water environments host diverse biocenoses with many potential predators, subterranean trophic chains are often topped by fewer intraguild predators (e.g., olms, planarians) (Dodds et al. 2019; Manenti et al. 2018; Romero 2020). In springs the situation is particularly complex as predatory fish, dytiscids, salamanders and dragonfly larvae can prey on both stygobiont and surface animals or affect their behavior (Manenti and Pezzoli 2019). For instance, the stygobiont crustacean *Niphargus thuringius* seems to exhibit lower spring exploitation when fire salamander ( *Salamandra salamandra* ) larvae are present because they may prey on it (Manenti, Forlani, et al. 2023).

Recent research suggested that springs can be more important than expected for stygobiont species (Manenti, Di Nicola, et al. 2024; Manenti and Piazza 2021; Gibson et al. 2008; Premate et al. 2022), still information on habitat exploitation and activity of stygobionts in springs is far from complete. Here we focused on atyid *Troglocaris planinensis*, an eyeless stygobiont shrimp of North‐eastern Italy (Manenti, Di Nicola, et al. 2024), and combined field surveys and behavioral tests to understand their response to the environmental variation occurring across ecotones. First, we evaluated in the field the factors favoring the exploitation of spring ecotones, assessing how predation risk and resource availability affect the presence and abundance of this highly mobile stygobiont shrimp. By recording the relative importance of spring features in explaining stygobiont shrimp abundance we aim to assess the existence of specific patterns in the exploitation of surface habitats by stygobionts. Second, we performed behavioral tests to compare responses to light and to chemical cues released by different predators (cave, surface and novel predators) between individuals of *T. planinensis* from cave and spring‐dwelling populations. This with the aim of depicting the existence of potential adaptations to ecotones exploitation by organisms considered as strictly bound in the subterranean realm.

## 2 Material and Methods

### 2.1 Study Species

The shrimp *Troglocaris planinensis* is a groundwater‐dwelling crustacean of the family Atyidae. The genus *Troglocaris* includes multiple morphologically similar species across the whole Dinaric region (Jugovic et al. 2011; Sket 1997). In the Italian part of the Dinaric Karst, two species of *Troglocaris* occur, namely *T. planinensis* and *T. anophtalmus* (Stoch 2017). Here we focused on *T. planinensis*, which is widely distributed in the area, while *T. anophtalmus* is reported only in two caves along the Isonzo‐Vipacco water system, which were excluded from this study (Jugovic et al. 2012).

*Troglocaris* shrimps are highly mobile, being able to swim in the water column and wander on the substrate. When disturbed, they can perform quick movements (Jugovic et al. 2010) and, in springs, they can quickly shelter back to groundwater crevices and holes (Manenti et al. 2025). *Troglocaris* shrimps are generally blind and eyeless. When analyzed under light‐, scanning‐, and transmission microscopy, adult individuals of the species *T. anophthalmus* showed no sign of ommatidia on the bare eyestalk or any related receptive structures (Meyer‐Rochow et al. 2011). Moreover, previous behavioral tests performed on four individuals of this species did not record any response to light stimuli (Meyer‐Rochow et al. 2011). Although it has been reported that the occasional occurrence of rudimentary retinal cell structures in some individuals of different species (Juberthie‐Jupeau 1972), only the subspecies *T. a. ocellata* regularly possesses pigmented eye rudiments (Jugovic et al. 2012), the functionality and adaptiveness of which are still to be understood.

*Troglocaris* shrimps are detritivores that consume organic matter from plants, animals, and microorganisms. Maximum longevity is about 10 years (Vogt 2012).

*Troglocaris* shrimps are often recorded in syntopy with the olm *Proteus anguinus* both in caves and springs (Jugovic et al. 2010; Manenti et al. 2025; Sket 1997). *P. anguinus* is usually the top predator in groundwater (Manenti et al. 2020), and predation on *Troglocaris* spp. individuals have been recorded (Briegleb 1963; Jugovic et al. 2010). Populations of *Troglocaris* spp. that co‐exist with *P. anguinus* seem to have substantially longer rostrums than those inhabiting aquifers without the olm (Jugovic et al. 2010); this could be considered an anti‐predatory defense, as according to the previously mentioned study it comes along with other adaptations such as enlarged carapaces and bulkier bodies (Jugovic et al. 2010).

### 2.2 Study Area

**FIGURE 1:**
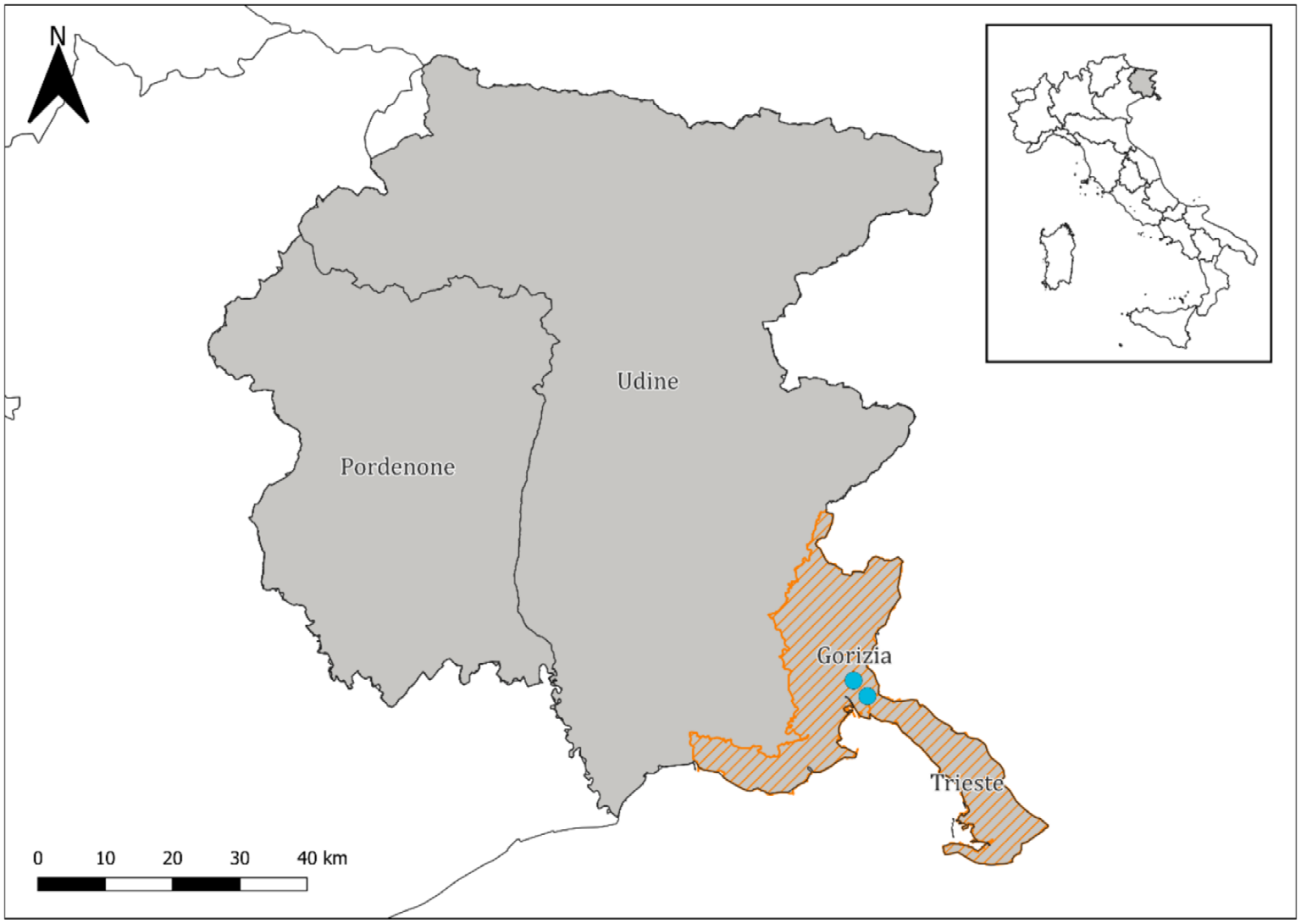
Location of the study area and sampling sites (light blue, approximated position) in Friuli‐Venezia Giulia. The monitored provinces are highlighted in orange.

The study area (Figure 1) is situated in the north‐western part of the Classical Karst, which is the northernmost part of the Eastern Adriatic karst territory (Jurkovšek et al. 2016). Most of the sampling sites, both springs and caves, were connected within a system of caves and karst lakes comprising Lake Doberdò, Lake Pietrarossa, Lake Sablici and Lake Mucille. The hydro‐ecological features of the study area encompass a wide variety of species and habitats (Castello et al. 2021) including caves (mostly vertical), springs, sinkholes and estavelles (i.e., openings that can function either as a spring or as a sink of freshwater).

### 2.3 Field Surveys

Between June 2020 and January 2025, we surveyed 64 spring sites multiple times (minimum: 2, maximum: 62, average ± SE = 13.71 ± 1.3). Surveys were carried out both during day and night. During each survey, we conducted 10‐min visual counts recording the number of *T. planinensis* individuals. During surveys, we recorded the number of potential predators, considering the olm *P. anguinus* and the pike (*Esox cisalpinus*), which is the top predator fish in the study system. We also recorded the occurrence of aquatic macrophytes, which are an index of productivity and can provide shelters and water flow level. Water flow was visually differentiated in four main categories: absent, occurring, moderate, strong. In limnocrene springs, we surveyed the whole spring mouths and estavelles openings. In case of fissures and other openings feeding rheocrene springs, we sampled the habitats for a maximum length downstream of 1 m.

### 2.4 Experimental Assays

In May 2022, we collected 100 individuals of *T. planinensis* from 5 sites (3 springs and 2 caves, *N* = 20 individuals per site) using dip nets (Table 1). In caves, sampling was conducted during the day, while in springs shrimp collection took place at dusk. Thermos flasks were adopted for transportation to keep the water temperature constant, reducing stress and mortality. After collection, the shrimps were temporarily housed in aquariums at the subterranean laboratory ‘Speleovivarium Erwin Pichl’ in Trieste, where they underwent a 7‐day acclimation period in large glass tanks (one per each sampling site), and two subgroups of 6–7 individuals for each sampling site were then transferred to 18 L plastic tanks. The animals spent 7 more days of acclimation in these housing tanks before the beginning of the experiments. Trickling water from the gallery complex was used as the water source for both the acclimation and the rearing tanks. Tanks had a constant source of water dripping via plastic tubing and an overflow drain at its top to maintain water exchange within each tank. Water temperature in the tanks ranged from 15.7°C to 16.2°C. Preliminary tests conducted with shrimps captured from “Abisso di Trebiciano” confirmed that *T. planinensis* is able to grow and reproduce in these conditions. Each tank was enriched with a thin layer of clay as substrate and some pebbles to mimic natural environments. Shrimps were fed around 2 pellets of Sera Shrimps Nature (lipids 7.7%, proteins 40.5%; Sera GmbH, Heisenberg, Germany) per individual every third day. Two days before the experiments, food provision was interrupted; residual pellets were periodically removed along with excrements and exuviae. Since a precise sex determination can be quite invasive for this species, we did not distinguish between females and males. Instead, we recorded the presence of eggs in the oviducts of females. Egg‐bearing females cannot represent females in general, but egg development appears to be a relatively long process that may allow to distinguish a certain number of females (Christodoulou et al. 2016).

**TABLE 1:** Location and date of collection of the shrimps of the species *Troglocaris planinensis* used in this study. Shrimps were released back in their collection sites at the end of the experiment.

Site of origin	Type of site	Collection date (day/month of 2022)
Doberdò del Lago	Spring	02/05
Abisso di Trebiciano	Cave	03/05
Pozzo presso S. Giovanni di Duino	C	04/05
Pietrarossa	Spring	05/05
Antro di Bagnoli	Spring	10/05

We performed two distinct experiments to assess the behavioral response variation between shrimps originating from different habitats (caves vs. springs). The first experiment assessed responsiveness to light stimuli; the second one assessed responsiveness to the chemical cues of potential predators.

**FIGURE 2:**
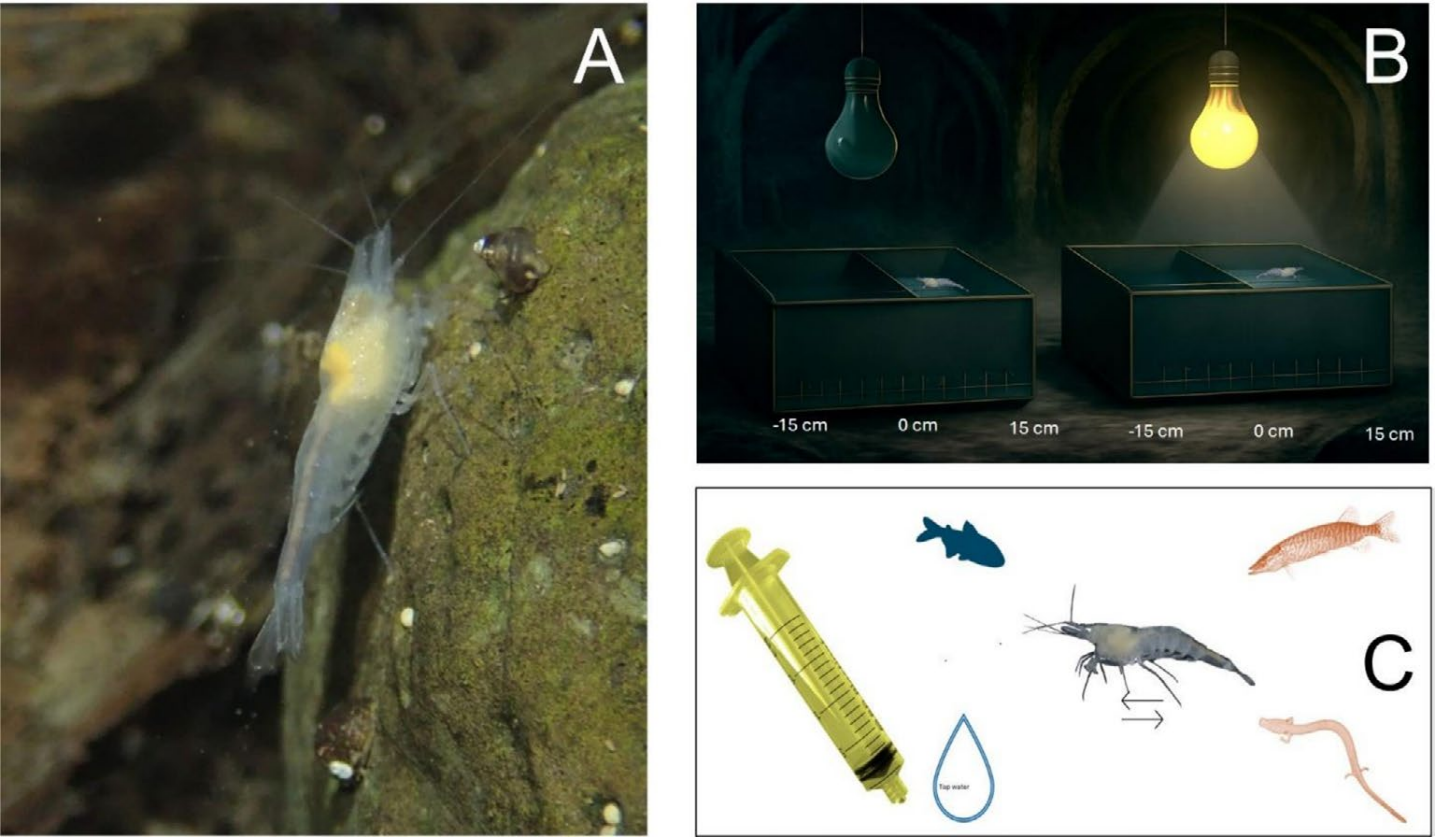
Methodological framework of the study. (A) An individual of the stygobiont shrimp *Troglocaris planinensis* observed during field survey. (B) Schematic of the experimental setting for testing responses to light stimuli by *T. planinensis* shrimps showing the key components and gothic‐inspired background representing the location (Speleovivarium of Trieste city) for esthetic effect generated with Chatbot AI. (C) Chemical cues used to assess behavioral responses to predators by *T. planinensis* (in the center); silhouettes in clockwise direction represent a Mexican tetra ( *Astyanax mexicanus* ), a pike (*Esox cisalpinus*), an olm ( *Proteus anguinus* ), and a drop of tap water.

The test assessing response to light was performed 7 days after acclimation in the final housing tanks following a protocol recently developed for stygobionts (Barzaghi et al. 2025). Three light conditions were tested: white light, red light, and light absence. Tests were performed in experimental arenas composed of a plastic container (30 × 15 × 5 cm) with a removable lid covering half of its surface to provide a shaded area on half of the experimental tank, regardless of the light condition being tested (Figure 2). The container was filled with 1‐L dechlorinated tap water that was changed after each individual was tested. A 30 cm ruler was placed parallel to the longer side of the experimental arena to measure shrimp position. The light source was a flashlight with different light settings; the distance between the light source and the experimental tank was kept at 122 cm. White light had an intensity of 25–50 lm, while red light averaged 25–40 lm. Two experimental stations were set up in different galleries with the same environmental conditions. The sequence of light conditions presented to the shrimps during the experiment was randomized. Logistical constraints hampered including individuals from “Antro di Bagnoli” in light exposure tests, and a total of 46 individuals from the remnant four sites (two from cave and two from springs) were tested in this experiment. During every experimental day, 12 animals (6 per housing tank) from each sampling site were individually measured, inspected for egg presence, photographed, and then put into either one of the two experimental arenas to be tested. Apart from Doberdò del Lago site (two individuals died.), during the experiment days a total of 12 individuals per site were tested.

After measuring their total length (TL), shrimps were individually placed into the test container in which they could freely roam during a 2‐min acclimation in total darkness. Tests consisted of a 5‐min test exposed to one of the three light conditions. After the 5 min of the test, the lid on the experimental arena was removed and animal position (distance between the position of the rostrum of the animal and the centre of the arena) was recorded. Negative values of shrimp position corresponded to preference for the dark side of the experimental arena, whereas positive values were associated with preference for the side exposed to light. Each animal underwent 6 consecutive tests (3 light treatments, 2 replicates each) in random order. Resting time between tests was of 5 min. At the end of the tests, individuals were brought back to their original housing tank and fed.

The experiment assessing responses to predator cues was performed seven days after the light one. Tests consisted in individual exposure to two different cues of predators occurring in the groundwater‐surface water system studied, such as the olm as subterranean predator and the pike as surface predator. Moreover, we added two control conditions such as a control without any cues (dechlorinated tap water) and a control for responses toward unknown chemicals using a predator unknown to *Troglocaris* shrimps, the Mexican tetra ( *Astyanax mexicanus* ). To obtain the cues, the predators were kept for 48 h without being fed as follows: an adult olm weighing 22.2 g placed in 2‐L water; a pike weighing 41.0 g kept in 4‐L of water; two tetras cumulatively weighing 24.0 g kept in 2.4 L of water. In this way, the biomass of all predators was around 10 g/L. From these containers, approximately 40 mL of water with chemical cue was transferred into 50 mL centrifuge tubes (10 tubes for each treatment) and stored frozen for later use. After chemical cue collection, the pike was released in its site of origin while olm and tetras were brought back to their respective housing tanks in the Speleovivarium. Two vials of each chemical cue were removed from the freezer and allowed to thaw at room temperature (16°C) for 24 h before each experimental session. Behavioral responses of shrimps to chemical treatments were conducted by placing individuals in experimental arenas and monitoring their behavior in 5‐min tests performed in total darkness. The arenas were squared plastic tanks measuring 20 × 20 cm, about 7 cm in height and filled with 1‐L dechlorinated tap water. Each experimental arena hosted one individual at a time during tests. We used IR cameras to record 12 arenas simultaneously. Overall, we tested 12 individuals of *T. planinensis* from every sampling site (six animals from each housing tank) except for Doberdò del Lago site from which we tested 10 individuals (total: 58 animals were tested, 24 from caves and 34 from springs). Before tests, shrimps were randomly placed in one of the available arenas and let acclimate for 20 min in dark conditions. The test started after the injection of 2 mL of the selected chemical cue in the centre of the arena and shrimp behavior was recorded in the subsequent five minutes. The same cue was used for all arenas at a time, while the order of chemical treatment exposure was random. Each individual underwent eight consecutive tests (4 chemical cue treatments, 2 replicates per treatment), separated by a 10‐min resting time. At the end of each experimental day, tested individuals were brought back to their original housing tank and fed. Survival during chemical cue experiments was 100%. Videos were analyzed with BORIS (Behavioral Observation Research Interactive Software), an open‐source software for ethological studies that allow the definition and recording of a set of standardized behavioral parameters (Friard and Gamba 2016). To assess shrimp responses to chemical treatments, we recorded the following behaviors:

- “Moving”: state event, used when the animal was found moving in the arena.
- “Standing”: state event used when the animal was found standing in the arena

Moving events were marked when the shrimp moved for at least half of its length and if the movement exceeded the threshold of 0.5 s; this allowed standardization of the measurement, while avoiding artifacts deriving from the impossibility to consistently quantify the duration of the micro‐movements. Nine videos were excluded because of the poor quality of recording. The final dataset was composed of 455 videos. Behavioral analyses with BORIS software were conducted by two trained operators (VZ, FC), randomly dividing videos between them. At the end of the experiments, the animals were released in their collection site.

### 2.5 Statistical Analyses

For the analysis of field data, we used a generalized linear mixed model (GLMM) (Bolker et al. 2008) to assess the relationships between the observed abundance of *T. planinensis* shrimps and environmental features. GLMMs produce reliable estimations of the relationships between the relative abundance of target species and environmental variation, providing estimates consistent with those obtained using alternative approaches such as N‐mixture models, and are particularly reliable in cases where the detection probability of individuals is relatively low (Barker et al. 2017). The number of shrimps individuals observed in each survey was the dependent variable, using a negative binomial error distribution. As fixed factors, we included the day period (day/night), the season, water flow level, and the occurrence of macrophytes, also including as covariates the number of olms observed and if at least a pike was observed during each survey. As a random effect, we used the identity of the site. We also tested the two‐way interactions between the day period and the number of pikes and olms. Non‐significant two‐way interactions were removed from the final model.

For the light experiment a Linear Mixed Model (LMM) (Bolker et al. 2008) was built. The dependent variable was the position of the individuals at the end of every test. As fixed factors, we selected the light treatment (white light, red light, light absence), the origin of the individuals (spring or cave), and if the individuals were ovigerous females or not. As random effects, we used the site of origin, the replicate of each treatment (first or second), and the identity of the shrimps. We also tested the two‐way interactions between the light condition and the origin of shrimps. Non‐significant two‐way interactions were not considered in the final model.

To assess responses to predator chemical cues, we built two LMMs. Basing on the behavioral parameters measured with BORIS (Friard and Gamba 2016), we selected two different dependent variables: “Total number of movements” performed and “Total time spent moving”. Both the two dependent variables were square‐rooted to improve normality, and for each of them we run identical models, selecting as fixed factors the origin of the individual (cave vs. spring), and the chemical cue treatment (control without cues tap water, control with unknown predator cues ‐Mexican tetra‐, cues of olm, cues of pike). As covariates we considered if individuals were ovigerous females and the trial number of each test (discrete variable from 1 to 8). As random effects we included, as for the light experiment, the site of origin, the replicate of each treatment (first or second) and the identity of the shrimps. We also tested the two‐way interactions between the chemical treatment and the origin of shrimps. Non‐significant two‐way interactions were not considered in the final model. As we detected a significant effect of the treatment on the number of movements performed by shrimps (see chapter results) we used Tukey's post hoc test to perform pairwise comparisons among all the four chemical cues used. Moreover, we used planned orthogonal contrasts (Field et al. 2015) to evaluate the effects of the two control conditions (no cues and unknown cues) by respect to the predator cues (olm and pike). To perform orthogonal contrasts, we replicated the model performed for the number of movements substituting the fixed factor ‘treatment’ with three contrasts: the first contrast tested whether the control treatments together differed from the predators' treatments together, the second one tested differences between water without cues and unknown tetra cues, and the third one differences between proteus and pike cues.

All the analyses were run in R v. 4.1.1 (R Development Core Team 2022) with packages *glmmTMB*, car, emmeans, *lme4* and *lmerTest*, and for graphs we used the packages *visreg* and *ggplot2* (Bates et al. 2014; Breheney and Burchett 2015; Brooks et al. 2017; Kuznetsova et al. 2017; Lenth 2025).

## 3 Results

### 3.1 Field Surveys

We recorded at least once the occurrence of *Troglocaris planinensis* in 47% of the sampled springs. Considering all the surveys, we counted on average (± SE) 1.68 (± 0.21) shrimps per survey. During the day we recorded on average 0.69 (± 0.14) shrimps, while during the night 2.23 (± 0.03) individuals. Abundance during the night was sometimes very high: in one case we detected 116 shrimps/m^2^ in a single spring mouth, while observations of 66.7 shrimps/m^2^, 64.1 shrimps/m^2^ and 34.5 shrimps/m^2^ occurred in other sites. During the day the maximum abundance observed was of 4.3 shrimps/m^2^ in a spring with a maximum illuminance of 142 lx; the highest illuminance at which we recorded shrimps in springs was of 5380 lx (density: 1.6 shrimps/m^2^). However, generally, no shrimps were recorded in patches directly exposed to sunlight, and the occurrence of *T. planinensis* was rare in sites with a maximum illuminance above 500 lx.

**FIGURE 3:**
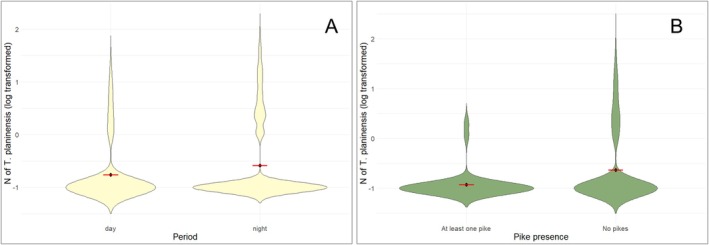
Main results of field surveys. (A) Violin plots showing the relationship between the number of *Troglocaris planinensis* observed in springs during field surveys and the period of the day (day or night). (B) Violin plots showing the relationship between the number of *Troglocaris planinensis* observed in springs during field surveys and the occurrence of pikes. The black points and the red lines show the mean value.

The GLMM confirmed that the number of shrimps was significantly higher during night (*χ* ^2^_1,937_ = 27.06, *p* < 0.001; Figure 3A). Moreover, the abundance of shrimps was negatively related to the presence of pikes (*χ* ^2^_1,866_ = 7.9, *p* < 0.01; Figure 3B, Figure S1) and to the water flow strength (*χ* ^2^_1,866_ = 14.74, *p* < 0.01). We also detected a slight effect of the season (*χ* ^2^_3,866_ = 8.66, *p* = 0.03). A subsequent post hoc Tukey test showed that it is likely to encounter more shrimps during summer than during winter (*z* = 2.9, *p* = 0.02). No effect of the abundance of olms (*χ* ^2^_1,866_ = 0.67, *p* = 0.18) nor of the occurrence of macrophytes (*χ* ^2^_1,866_ = 0.01, *p* = 0.97) was observed (Figure S1).

### 3.2 Behavioral Responses

**FIGURE 4:**
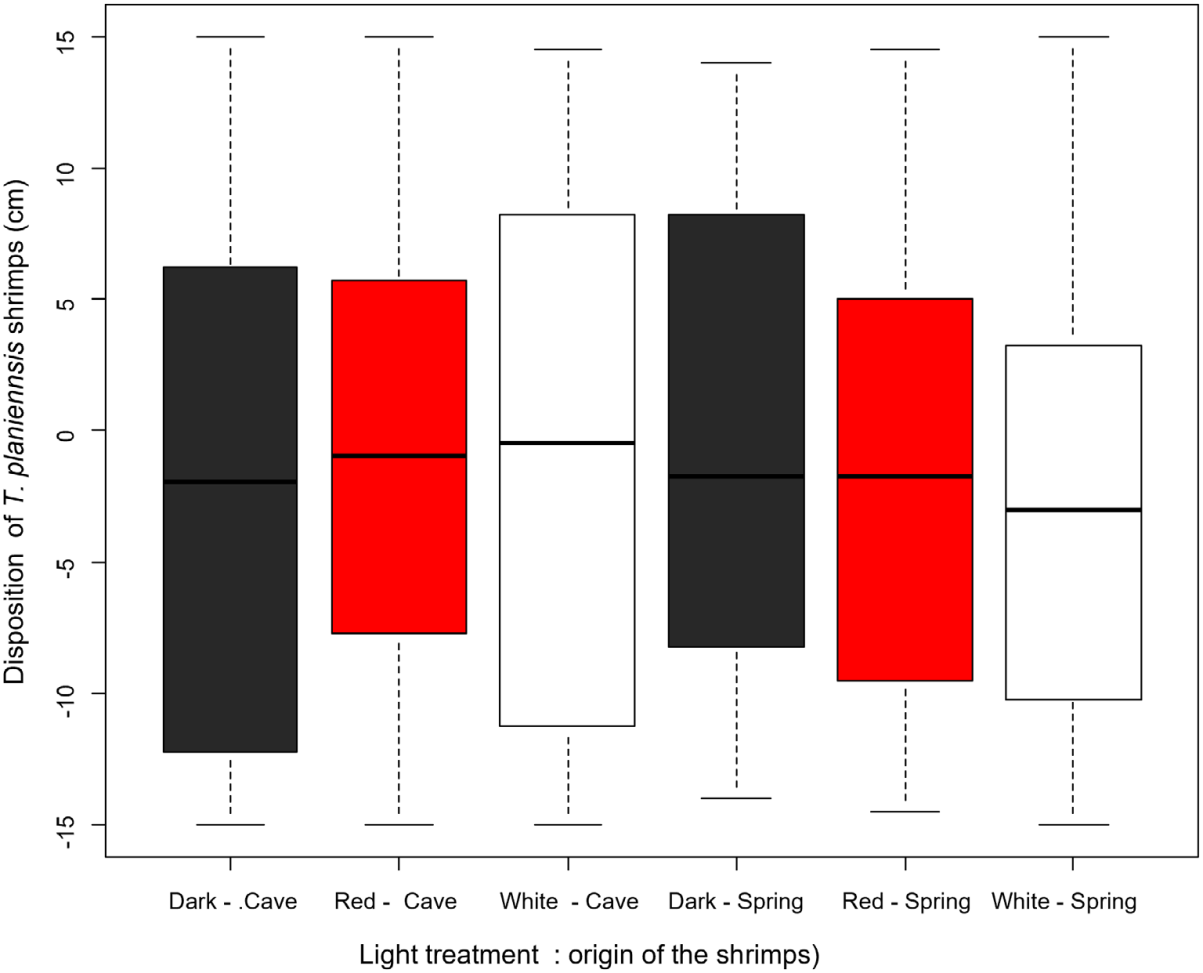
Relationships between light treatments and position of shrimps in the experimental arenas, according to the origin (cave or spring) of collection. Negative values indicate dark, covered side of the arena, while positive light the side not covered by lid and illuminated according to the treatment. Black boxplots show treatment without light stimuli, red boxplots treatment with red light and white boxplots treatment with white light stimuli.

Out of the 46 *Troglocaris planinensis* individuals included in the light experiment 14 were ovigerous females. The mean ± SD individual total length was 26.7 ± 3.4 mm. Overall, the average position at the end of the light test was −1.9 cm (SE = 1 cm) under white light, −1 cm (SE = 1 cm) under red light, and −0.9 cm (SE = 0.9 cm) in darkness (Figure 2). The LMM did not detect significant effects of light treatment (*F*_2,226.19_ = 0.37, *p* = 0.658, Figure 4, Figure S2) nor of the origin (*F*_1,2.15_ = 0.01, *p* = 0.944, Figure S2) on shrimp position in the experimental arena. Also, the effect on ovigerous females was not significant *F*_1,44.01_ = 0.11, *p* = 0.734.

**FIGURE 5:**
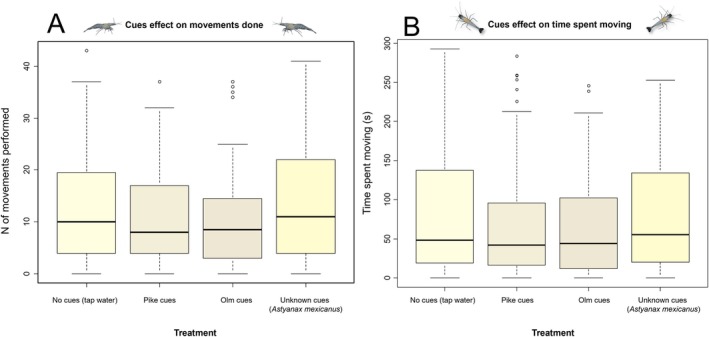
Box‐whiskers plots showing (A) the relationship between the number of movements performed by *Troglocaris planinensis* shrimps and (B) the relationship between the time spent moving by *T. planinensis* shrimps and the chemical cues used during behavioral experiments.

The mean ± SD total length of the 58 shrimps used for the experiment testing responses to chemical cues was 26.7 ± 3.3 mm, and 17 of them showed eggs. During tests, the overall number of movements performed was on average ± SE of 10.12 ± 0.8 with olm cues, 10.79 ± 0.79 with pike cues, 12.18 ± 0.95 in the control treatment without cues and of 13.38 ± 1.01 in the control treatment with the unknown predator cues (Figure 5A). Shrimps from springs performed on average ± SE of 10.58 ± 0.55 movements while shrimps from caves 13.08 ± 0.72 movements. The mean ± SE time spent moving by shrimps was 74.06 ± 3.36 s, moving on average 65.63 ± 5.79 s with olm cues, 68.16 ± 6.3 s with pike cues and 78.67 ± 7.11 s and 84.07 ± 7.16 s with no cues and unknown cues respectively (Figure 5B).

LMMs revealed that the total number of movements was significantly affected by the treatment (Table 2; Figure S3). Pairwise comparisons showed that *T. planinensis* moved significantly less with olm cues than with unknown cues (*z* = 2.85, *p* = 0.02, Table S1) and orthogonal contrasts revealed that if we consider predators cues together (olm and pike), shrimps moved significantly less than with control cue treatments (*F*_1_ = 6.31, *p* = 0.01; Table S2; Figure S4). Moreover, the number of movements was affected also by the number of tests done with shrimps showing a general decrease of the number of movements as the number of tests undertaken increased (Table 2). We did not detect any effect of the origin of *T. planinensis* individuals (spring or cave) nor of being ovigerous females (Table 2).

**TABLE 2:** LMM results for the total number of movements (transformed using square root) that Troglocaris planinensis individuals performed during experiments with chemical cues.

Variables	Estimate	df	*F*	*p*
Origin (spring)	−0.41	1, 3.01	0.68	0.47
Treatment		3, 401.06	2.84	**0.03**
Ovigerous females	0.17	1, 54.98	0.37	0.54
Number of tests done	−0.08	1, 401.01	10.53	**< 0.01**

*Note:* Bold values represent significant results.

LMMs did not detect any effect of the origin of the shrimps (cave or surface) nor of the treatments on time spent moving (Table 3, Figure S5). Again, a significant and negative effect of the number of tests done was recorded (Table 3).

**TABLE 3:** LMM results for the total time moving (transformed using square root) that Troglocaris planinensis individuals spent moving during experiments with chemical cues.

Independent variable	Estimate	DF	*F*	*p*
Origin (spring)	−0.93	1, 2.98	0.51	0.52
Treatment		3, 401.07	1.94	0.12
Ovigerous female	−0.09	1, 55	0.01	0.89
Number of tests done	−0.33	1, 401	19.63	**< 0.01**

*Note:* Bold values represent significant results.

## 4 Discussion

### 4.1 Role of Diel Variation and Springs' Features in Allowing Their Exploitation

Field surveys showed a relevant presence and abundance of the shrimp *Troglocaris planinensis* in springs. Almost half of the sites investigated are used by the species, which can reach high densities, especially during night, suggesting that they can constitute a noteworthy portion of the overall biomass of macroinvertebrates of some springs. Notably, our findings document a significant diurnal variation in activity levels; shrimps were significantly more abundant during nocturnal hours, even if some individuals were recorded also during sunny days. This corroborates the findings reported for other stygobiont species including the olm (Manenti, DI Nicola, et al. 2024; Premate et al. 2022), crustaceans of the genus *Niphargus* (Kureck 1967; Manenti and Barzaghi 2021) and eyeless planarians of the genus *Dendrocoelum* (Barzaghi et al. 2025). The retention or loss of surface‐related traits in cave fauna is largely contingent upon evolutionary trade‐offs that balance energetic expenditure with the adaptive advantages associated with them (Friedrich 2013, 2024; Moran et al. 2014). Caves represent dark environments that are usually strongly depleted of trophic resources, thereby favoring the loss of non‐functional traits that impose high metabolic costs as a consequence of natural selection (Moran et al. 2014, 2015; Romero 2020). Consequently, the reduction of complex visual systems in species that inhabit groundwater stands as one of the most prevalent adaptations (Niemiller et al. 2016; Pipan and Culver 2012; Trontelj et al. 2012). Nonetheless, selection might favor the preservation of simpler structures that facilitate light detection, such as extraocular photoreceptors (Brodrick and Jékely 2023), provided they confer some functional benefits. Photophobia in stygobionts is commonly associated to the relevance of perceiving borders between groundwater and surface (Fišer et al. 2016). For instance, this should allow *Niphargus* amphipods to avoid risky surficial environments (Fišer 2019; Fišer et al. 2014, 2016). Recently this capability has also been hypothesized to provide an adaptive advantage to exploit springs and surface freshwater when they are less risky, such as during night (Manenti and Barzaghi 2021; Manenti and Piazza 2021). Despite the higher number of *T. planinensis* observed in springs at night could in principle lead to hypothesize that a similar pattern exists also for this species, our behavioral experiment did not detect significant responses of shrimps to light, confirming previous behavioral tests performed on few individuals of *T. anophtalmus* (Meyer‐Rochow et al. 2011). This may be linked to the fact that to perceive diel variation in springs this species uses different cues than the ones here tested.

Consistent with this possibility, field surveys showed that *T. planinensis* was more abundant at sites with lower water flow, suggesting a preference for lentic conditions and possible avoidance of drift risk in surface habitats. Moreover, we detected a difference between the number of individuals occurring in winter and in summer. Future research is thus needed to test the role of alternative cues that can determine the day‐night activity pattern, such as UV light, variations in temperature, chemicals associated with variations in water flow, mechanical stimuli, or nutrients. Variation in abundance of stygobiont shrimps between night and day could be also linked to the diel activity of other species, especially visually driven fish predators, as observed in other atyid crustaceans (Capps et al. 2009; Carey et al. 2011; Havird et al. 2013).

### 4.2 Perception of Surface Predators

Our laboratory experiments revealed that *T. planinensis* shrimps can respond to chemical cues of predators suggesting that multiple cues may be involved in shaping patterns of surface habitats exploitation. Shrimps exhibited fewer movements in response to predator cues compared to control stimuli. Although *T. planinensis* did not differentiate distinctly between olm and pike cues, both affected its behavior. The olm cues had a prominent impact, but responses to pike cues were similar, indicating the shrimp's ability to recognize these stimuli. Notably, movement duration showed no treatment effect, suggesting a need for more trials or further analysis to uncover underlying behavioral patterns. Variations in movement number, duration, and speed may be influenced by factors such as adaptations, stimuli (e.g., food cues), and environmental conditions (Azarm‐Karnagh et al. 2023; De Tailly et al. 2025; Hanson et al. 2023). When predators detect prey via thigmotactic and vibrational cues—or via vision under low‐light conditions—reducing movement frequency may, in theory, offer some survival and foraging advantage than merely minimizing movement time (Glaudas and Alexander 2017; Sousa et al. 2011). Infrequent, movements limit cue production, whereas slow, continuous movement can generate detectable signals for visual predators. This strategy can be especially beneficial for mobile prey, which rely on rapid movements for risk avoidance and retreat to shelters when disturbed. Previous observations revealed that in springs, *T. planinensis* individuals can rapidly retreat to groundwater when disturbed (Manenti et al. 2025). It is thus possible that, together with chemical stimuli, variation in water vibrations and other mechanotactic cues may contribute to modulating spring habitat exploitation of this species. Finally, it is noteworthy that in the subspecies *T. anophtalmus ocellata*, which is often recorded in spring habitats, different individuals maintain pigmented eye rudiments (Jugovic et al. 2012). It could be thus interesting replicating light exposure tests in *T. a. ocellata* individuals with and without eyes.

Diel activity is not the only factor that seems to affect the abundance of *T. planinensis* in the field, as we detected a significant and negative effect of the pike presence in springs. The pike is a typical surface predator, and its presence has never been documented within the cave systems of the study area (Stoch 2017), but pikes can migrate upstream toward spring mouths and estavelles for breeding, and juveniles can be fairly common in these environments (Chizinski et al. 2016; Gurbik et al. 2015; Pompei et al. 2017; Rittweg et al. 2024; Walton‐Rabideau et al. 2020). Field records partially support the hypothesis that predator–prey interactions and predation dynamics can play a role as a regulatory mechanism within the spring ecosystems (Manenti, Forlani, et al. 2023). Juvenile pikes can prey upon different macroinvertebrates and it is likely that in springs prey can include *T. planinensis*. Even if in laboratory conditions the main response that we detected was induced by olm cues, considering together both olm and pike cues drove a significant reduction of movement compared to control conditions. The olm is the main predator of *Troglocaris* shrimps in groundwaters, and populations co‐existing with it evolved specific adaptations like longer rostra armed with more numerous teeth to avoid predation (Jugovic et al. 2010). Taken together, our field and laboratory results suggest that *T. planinensis* likely recognize pike and/or other surface predators and modulate their behavior accordingly, regardless of the origin of populations (spring or cave). These results provide a first, empirical evidence that a typical stygobiont species perceives and responds to stimuli originating in spring habitats. Shrimps adjusted their activity in laboratory conditions and in the exploitation of the surface environment, demonstrating behavioral flexibility beyond strictly subterranean constraints. This ability to cope with epigean cues can have implications for exposure to surface‐associated risks and resources. Nonetheless, observations performed during both surveys and experiments hint that *Troglocaris* shrimps are highly mobile, often performing brief or longer swimming movements along the water column and crawling in the detritus in search of organic matter. This suggests that more behavioral traits and additional factors should be studied to measure the responses that can help *Troglocaris* shrimps escape predators in the wild and that can allow them to distinguish groundwater from springs. For instance, both shelters, thigmotactic elements, or the presence of trophic resources were absent in our experimental settings and this can have influenced shrimps' responses. Indeed, shelter‐seeking behaviors and variations in diel activity with preference for night hours have been evidenced to be common antipredator responses toward predatory fish in epigean atyid and palaemonid shrimps (Capps et al. 2009; Lopez et al. 2016; Silva et al. 2024). Future studies could consider investigating the role of similar factors in shaping *T. planinensis* behavior.

### 4.3 Effect of the Origin of the Shrimps and Caveats

Studying blind prey shrimp from both groundwater (where predators cannot rely on visual stimuli) and springs (where both blind and visual predators occur) can be important for research aiming at understanding adaptive divergence between cave and surface habitat, a topic explored till now mainly with isopod and amphipod crustaceans (Biró et al. 2024). However, in our study we did not detect behavioral differences between shrimps from caves and springs. Different studies suggest that cave‐dwelling animals, especially in the early phase of cave colonization, might demonstrate heightened behavioral plasticity compared to surface‐dwelling counterparts (Bendik et al. 2013; Manenti et al. 2016; Pacioglu et al. 2020; Romero 2011). This was not the case with our study populations. A limitation of the experiment is the short testing duration (5 min), as shrimps—particularly individuals originating from caves—may not have fully acclimated to the arena despite the relatively long acclimation period. Future studies could apply the same experimental design over longer observation periods. Finally, also the observation that shrimps tended to move less in the later trials deserves a comment. It might be related to stress or to habituation. In case of stress, such effect might be prevented with a longer period of acclimation between each test, even if this would imply a bigger expense in terms of time and resources (Ginet 1960). Habituation can happen to *T. planinensis* shrimps when continuously receiving chemical cues of predators in harmless conditions as observed for other caridean species (Ocasio‐Torres et al. 2021). Patterns favoring or contrasting the overreacting to chronic stimuli (Hubert et al. 2022; Lima and Bednekoff 1999) could be investigated in the future.

## 5 Conclusions

In conclusion, our study lays the basis for an in‐depth investigation of two primary hypotheses that remain unsolved. First (i), the connections between groundwater and springs can be, in general, quite strong from a biological perspective, suggesting more frequent movements than previously thought of individuals of stygobiont species from inner areas to surface and vice versa. Generally, stygobiont species are reported to perform limited movements (Balázs et al. 2020; Gutierrez et al. 2018; Malard et al. 2023), even if for predatory species a wide wandering strategy has been reported (Manenti, Vinci, et al. 2024; Uiblein et al. 1992, 1995). There is evidence that specialization to stable subterranean habitats may be selected against dispersal because stenoecious organisms could encounter lethal/stressful conditions during wide displacements (Culver and Pipan 2011; Malard et al. 2023). However, *T. planinensis* shrimps are quite mobile and, during our five‐year long field survey, we detected strong variations in water level of springs. It is evident that *T. planinensis* can follow the variation of the water table, which can vary between tens of meters (Zini et al. 2022). Moreover, the use of springs by shrimps can be only intermittent and limited to short periods of high‐water levels, at least in some of the sites that we considered. Second (ii), we can still consider the hypothesis that the differing pressures and advantages presented by groundwater versus surface waters could drive, at least in some conditions, significant adaptations and differentiation among species residing in springs and caves. Even with ongoing gene‐flow, differences can be promoted between contiguous and shallow environments (Manenti, Kristensen, et al. 2023; Stern et al. 2017; Storfer et al. 1999; Velo‐Anton et al. 2012).

Thus, a pressing question that requires further examination is whether individuals of *T. planinensis*—and potentially other stygobiont species—can perceive the distinct features of subterranean and surface habitats. Moreover, it could be particularly interesting to investigate the potential responses to water flow variation, which can pose a strong pressure as once drifted far from the spring mouth, stygobiont shrimps can encounter a strongly unfavorable habitat with low possibilities of survival (Dole‐Olivier et al. 1997). Also, temperature variations acting in springs and epigean habitats at the border of the continuum of stygobionts occurrence can drive differences among individuals/sub‐populations that more or less regularly exploit the ecotone (Kreiling et al. 2022).

Our study, by integrating both field and laboratory approaches, adds further knowledge on the behavioral ecology of stygobiont shrimps, highlighting the interplay between diel activity and predator avoidance in shaping the exploitation of spring ecosystems. Our results also underscore the need for further investigation into the specific cues and behavioral mechanisms acting along the groundwater‐surface ecotone.

## Author Contributions

**Raoul Manenti:** conceptualization (lead), data curation (equal), formal analysis (equal), funding acquisition (lead), methodology (equal), writing – original draft (equal). **Veronica Zampieri:** data curation (equal), investigation (lead), methodology (equal), writing – original draft (equal). **Filippomaria Cassarino:** investigation (equal), methodology (equal). **Damiano Brognoli:** data curation (equal), investigation (equal), writing – review and editing (equal). **Edgardo Mauri:** methodology (equal), resources (equal), supervision (equal), writing – review and editing (equal). **Giorgia Terraneo:** investigation (equal), writing – review and editing (equal). **Elia Lo Parrino:** investigation (equal), validation (equal), writing – review and editing (equal). **Raffaele Bruschi:** investigation (equal), resources (equal), writing – review and editing (equal). **Valentina Balestra:** investigation (equal), resources (equal), writing – review and editing (equal). **Stefano Lapadula:** investigation (equal), writing – review and editing (equal). **Matteo Galbiati:** investigation (equal), writing – review and editing (equal). **Valeria Messina:** investigation (equal), writing – review and editing (equal). **Mattia Falaschi:** formal analysis (equal), writing – review and editing (equal). **Benedetta Barzaghi:** investigation (equal), methodology (equal), resources (equal). **Gentile Francesco Ficetola:** formal analysis (equal), methodology (equal), writing – review and editing (equal). **Andrea Melotto:** formal analysis (lead), writing – original draft (equal).

## Funding

This work was supported by Ministero dell'Università e della Ricerca, PRIN 2022 STIGE‐CLIMAQUIFERI DTA.PN010.014 2022MM8.

## Conflicts of Interest

The authors declare no conflicts of interest.

## Supporting information


**Table S1:** Results of pairwise comparisons between treatments with different chemical cues to assess their role in affecting the total number of movements (transformed using square root) that *Troglocaris planinensis* individuals performed during experiments.
**Table S2:** Effects of predator cues on the movements of *Troglocaris planinensis* shrimps. LMM results for the total number of movements that *Troglocaris planinensis* performed during experiments considering orthogonal contrasts between the treatment conditions tested.
**Figure S1:** (A–F) Results of the GLMM for the field surveys on the number of *Troglocaris planinensis* observed in springs during day and during night; effect of pike presence (A), olm abundance (B), day period (night ‐day) (C), macrophytes occurrence (D), season (E) and year of survey (F). The gray boxes represent 95% confidence interval, the light blue line represents the median, and the dots represent the residuals estimated by the model.
**Figure S2:** (A–C) Results of the LMM for position of *Troglocaris planinensis* during experimental tests on reaction to light stimuli, according to (A), origin of the shrimps (spring or cave) (B), light treatment and (C) if they were or not ovigerous females. The gray boxes represent 95% confidence interval, the light blue line represents the median, and the dots represent the residuals estimated by the model.
**Figure S3:** (A–D). Results of the LMM for the total number of movements performed by *Troglocaris planinensis* during experimental tests with the chemical cues of potential predators, effect of the origin (A), treatment (B), if individuals were or not ovigerous females (C) and number of tests done (D). The gray boxes represent 95% confidence interval, the light blue line represents the median, and the dots represent the residuals estimated by the model.
**Figure S4:** (A–E) Results of the LMM for the total number of movements performed by *Troglocaris planinensis* during experimental tests with the chemical cues of potential predators considering orthogonal contrasts between the treatment conditions tested, effect of the origin (A), of predator cues vs. control conditions (B), of olm cues vs. pike cues (C), of control without chemical cues vs. control with unknown cues (D), of the fact that individuals were or not ovigerous females (E) and of the number of tests done (F). The gray boxes represent 95% confidence interval, the light blue line represents the median, and the dots represent the residuals estimated by the model.
**Figure S5:**. (A–D) Results of the LMM for the total time spent moving by *Troglocaris planinensis* during experiments assessing the effects of the chemical cues of potential predators, effect of origin of the individuals (A), treatment (B), if individuals were or not ovigerous females (C) and number of tests done (D). The gray boxes represent 95% confidence interval, the light blue line represents the median, and the dots represent the residuals estimated by the model.

## Data Availability

Data are available in figshare at: https://doi.org/10.6084/m9.figshare.29994250.v1. Analyses reported in this article can be reproduced using the data provided.
