# Supplementary material for: Living at the Interface: Behavioral, Evolutionary and Ecological Insights of Spring Use by Highly Mobile Stygobiont Crustaceans, Troglocaris planinensis (Decapoda: Atyidae)
Source: Ecol Evol. 2026 Mar 27;16(4):e73338. doi: 10.1002/ece3.73338 (PMC13107282; doi:10.1002/ece3.73338)
Supplement: Supplementary file 1 — Table S1: Results of pairwise comparisons between treatments with different chemical cues to assess their role in affecting the total number of movements (transformed using square root) that Troglocaris planinensis individuals performed during experiments. Table S2: Effects of predator cues on the movements of Troglocaris planinensis shrimps. LMM results for the total number of movements that Troglocaris planinensis performed during experiments considering orthogonal contrasts between the treatment conditions tested. Figure S1: (A–F) Results of the GLMM for the field surveys on the number of Troglocaris planinensis observed in springs during day and during night; effect of pike presence (A), olm abundance (B), day period (night ‐day) (C), macrophytes occurrence (D), season (E) and year of survey (F). The gray boxes represent 95% confidence interval, the light blue line represents the median, and the dots represent the residuals estimated by the model. Figure S2: (A–C) Results of the LMM for position of Troglocaris planinensis during experimental tests on reaction to light stimuli, according to (A), origin of the shrimps (spring or cave) (B), light treatment and (C) if they were or not ovigerous females. The gray boxes represent 95% confidence interval, the light blue line represents the median, and the dots represent the residuals estimated by the model. Figure S3: (A–D). Results of the LMM for the total number of movements performed by Troglocaris planinensis during experimental tests with the chemical cues of potential predators, effect of the origin (A), treatment (B), if individuals were or not ovigerous females (C) and number of tests done (D). The gray boxes represent 95% confidence interval, the light blue line represents the median, and the dots represent the residuals estimated by the model. Figure S4: (A–E) Results of the LMM for the total number of movements performed by Troglocaris planinensis during experimental tests with the chemical cues of potential [file ECE3-16-e73338-s001.docx]

Supplementary information for the paper titled” Living at the interface: behavioural, evolutionary and ecological insights of spring use by highly mobile stygobiont crustaceans”

**Supplementary tables**

| **Comparison** | **Estimate** | **DF** | **t** | **P** |
| --- | --- | --- | --- | --- |
| Control without cues (tap water) - pike | 0.12 | 401 | 0.72 | 0.88 |
| Control without cues (tap water) - olm | 0.27 | 394 | 1.61 | 0.36 |
| Control without cues (tap water) – Control with unknown cues (*Astyanax mexicanus*) | -0.19 | 376 | -1.13 | 0.67 |
| Pike - Olm | 0.15 | 401 | 0.91 | 0.79 |
| Pike - Control with unknown cues (*Astyanax mexicanus*) | -0.31 | 396 | -1.87 | 0.24 |
| Olm- Control with unknown cues (*Astyanax mexicanus*) | -0.46 | 400 | -2.82 | **0.02** |

**Supplementary Table 1.** Results of pairwise comparisons between treatments with different chemical cues to assess their role in affecting the total number of movements (transformed using square root) that Troglocaris planinensis individuals performed during experiments.

| **Variable** | **Estimate** |  | **DF** | **F** | P |
| --- | --- | --- | --- | --- | --- |
| Origin (spring) | -0.93 |  | 1, 2.98 | 0.51 | 0.52 |
| Predators cues vs controls cues | -0.18 |  | 1, 401.07 | 4.86 | **0.02** |
| Olm cues vs pike cues | -0.08 |  | 1, 401 | 0.1 | 0.75 |
| Control without cues – Control with unknown cues | -0.22 |  | 1, 401.14 | 0.86 | 0.35 |
| Ovigerous females | -0.09 |  | 1, 55 | 0.01 | 0.89 |
| Number of tests done | -0.33 |  | 1, 401 | 19.63 | **< 0.01** |

**Supplementary Table 2.** Effects of predator cues on the movements of Troglocaris planinensis shrimps. LMM results for the total number of movements that Troglocaris planinensis performed during experiments considering orthogonal contrasts between the treatment conditions tested.

**Supplementary figures**

**Supplementary Figure 1. (A-F)** Results of the GLMM for the field surveys on the number of Troglocaris planinensis observed in springs during day and during night; effect of pike presence (A), olm abundance (B), day period (night -day) (C), macrophytes occurrence (D), season (E) and year of survey (F). The grey boxes represent 95% confidence interval, the light blue line represents the median, and the dots represent the residuals estimated by the model.


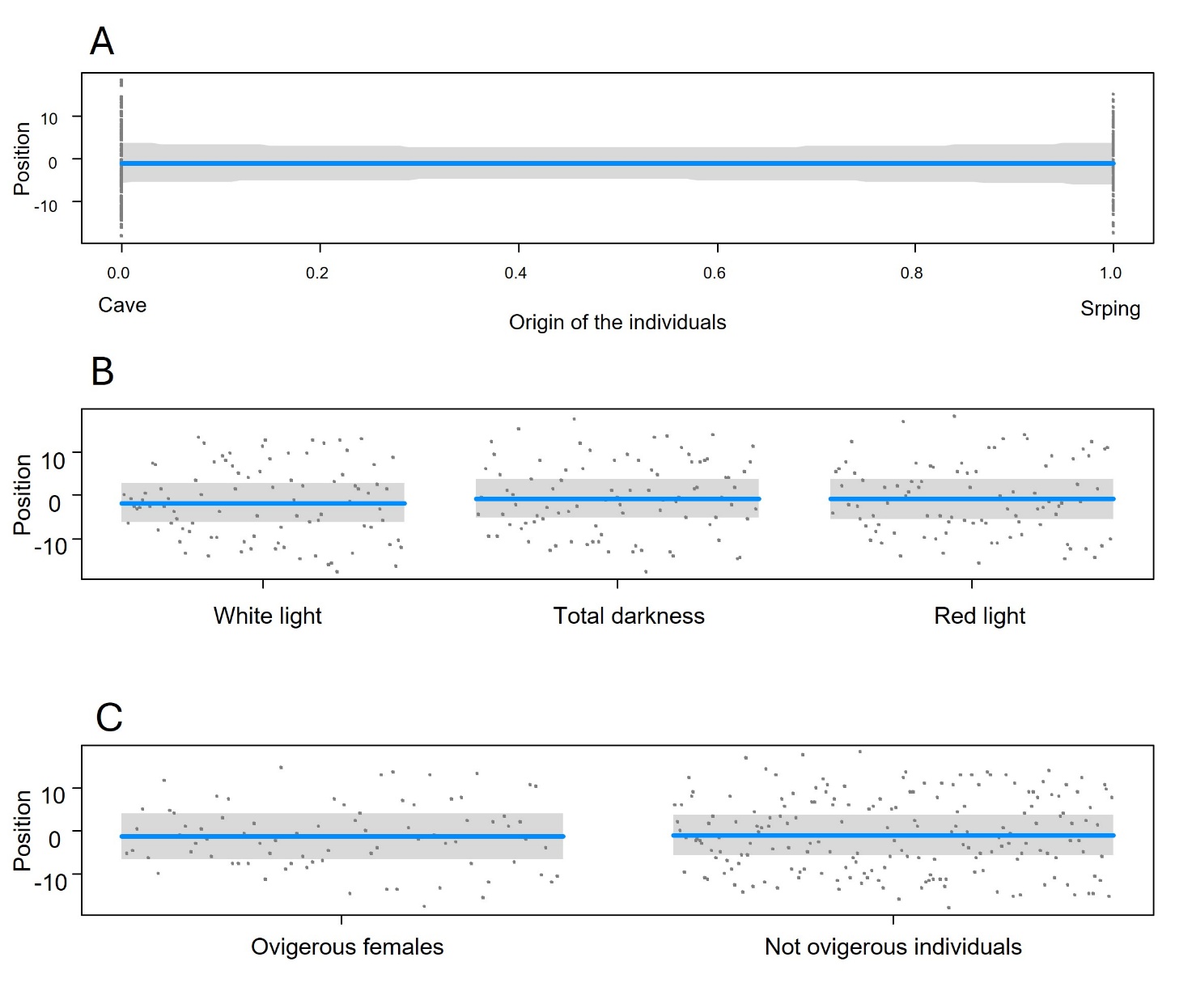


**Supplementary Figure 2 (A-C)** Results of the LMM for position of Troglocaris planinensis during experimental tests on reaction to light stimuli, according to (A), origin of the shrimps (spring or cave) (B), light treatment and (C) if they were or not ovigerous females. The grey boxes represent 95% confidence interval, the light blue line represents the median, and the dots represent the residuals estimated by the model.


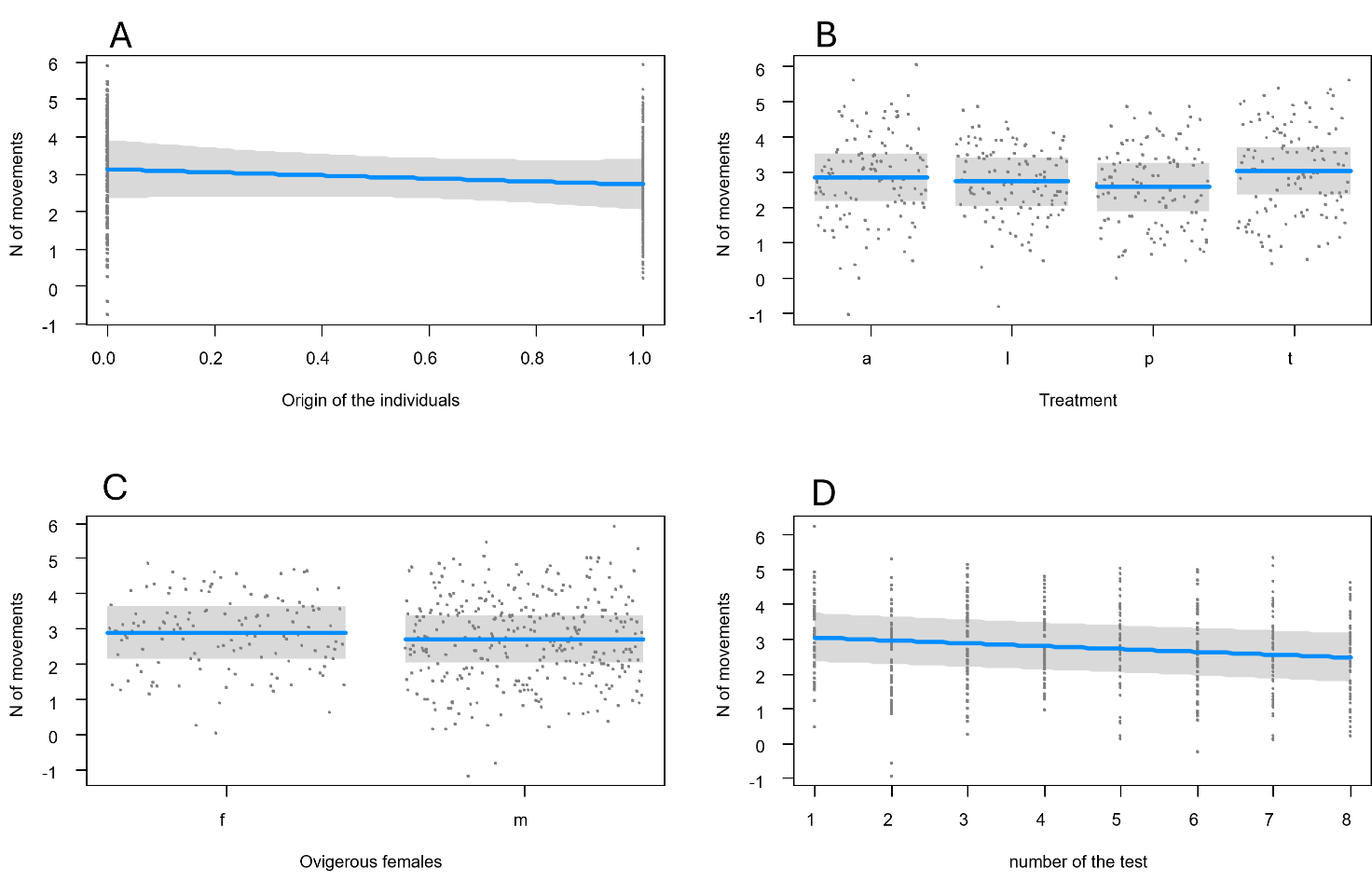


**Supplementary Figure 3 (A-D).** Results of the LMM for the total number of movements performed by Troglocaris planinensis during experimental tests with the chemical cues of potential predators, effect of the origin (A), treatment (B), if individuals were or not ovigerous females(C) and number of tests done (D). The grey boxes represent 95% confidence interval, the light blue line represents the median, and the dots represent the residuals estimated by the model.


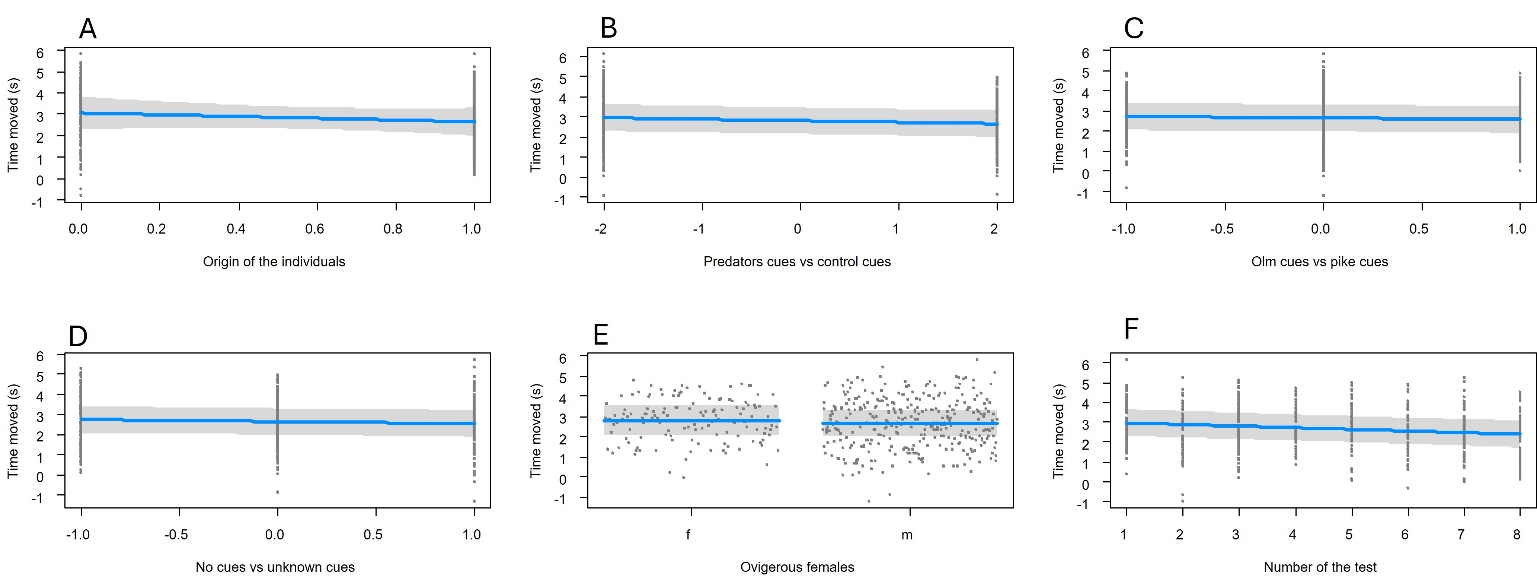


**Supplementary Figure 4 (A-E)** Results of the LMM for the total number of movements performed by Troglocaris planinensis during experimental tests with the chemical cues of potential predators considering orthogonal contrasts between the treatment conditions tested, effect of the origin (A), of predator cues vs control conditions (B), of olm cues vs pike cues (C), of control without chemical cues vs control with unknown cues (D), of the fact that individuals were or not ovigerous females (E) and of the number of tests done (F). The grey boxes represent 95% confidence interval, the light blue line represents the median, and the dots represent the residuals estimated by the model.


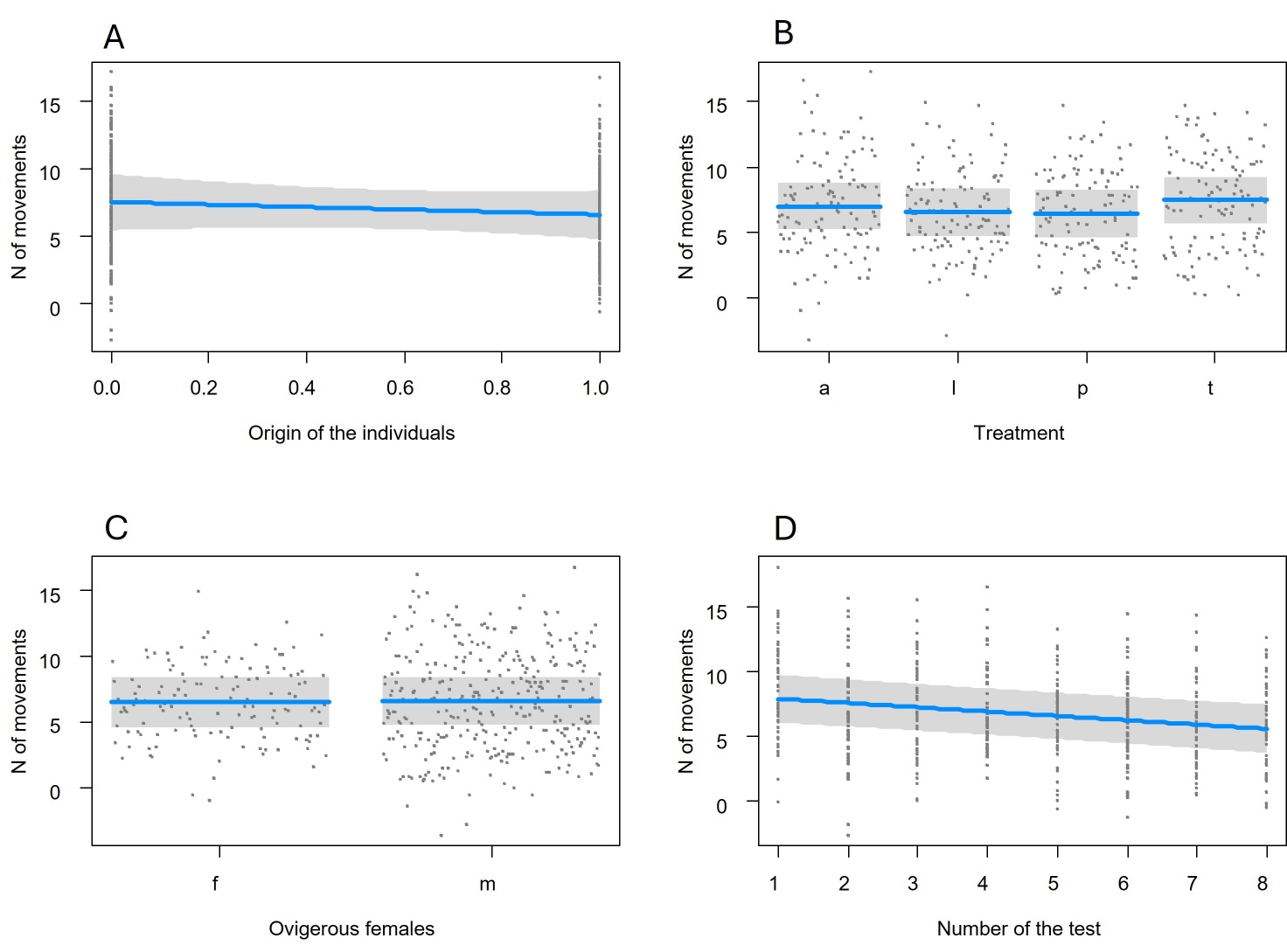


**Supplementary Figure 5 (A-D)** Results of the LMM for the total time spent moving by Troglocaris planinensis during experiments assessing the effects of the chemical cues of potential predators, effect of origin of the individuals (A), treatment (B), if individuals were or not ovigerous females (C) and number of tests done (D). The grey boxes represent 95% confidence interval, the light blue line represents the median, and the dots represent the residuals estimated by the model.
